# Comparative Analysis of Diverse Acetyltransferase-Type Toxin-Antitoxin Loci in Klebsiella pneumoniae

**DOI:** 10.1128/spectrum.00320-22

**Published:** 2022-06-15

**Authors:** Ying-Xian Goh, Peifei Li, Meng Wang, Marko Djordjevic, Cui Tai, Hui Wang, Zixin Deng, Zhaoyan Chen, Hong-Yu Ou

**Affiliations:** a State Key Laboratory of Microbial Metabolism, Joint International Laboratory on Metabolic & Developmental Sciences, School of Life Sciences & Biotechnology, Shanghai Jiao Tong University, Shanghai, China; b Intensive Care Unit, First Affiliated Hospital of Guangxi Medical University, Nanning, Guangxi Province, China; c Quantitative Biology Group, Institute of Physiology and Biochemistry, Faculty of Biology, University of Belgrade, Belgrade, Serbia; d State Key Laboratory of Pathogens and Biosecurity, Beijing Institute of Microbiology and Epidemiology, Beijing, China; South China Sea Institute Of Oceanology

**Keywords:** toxin and antitoxin system, GNAT and RHH, acetyltransferase, *Klebsiella pneumoniae*

## Abstract

Toxin-antitoxin (TA) modules containing a Gcn5-related *N*-acetyltransferase (GNAT) toxin domain regulate bacterial physiology under adverse environmental stresses. Multiple GNAT-ribbon-helix-helix domain (RHH) TA loci have been identified in single bacterial genomes. However, their diversity and interactions are still obscure. Our previous analysis showed that the GNAT toxin of Klebsiella pneumoniae, KacT, introduces antibiotic tolerance and the toxicity of GNAT is neutralized by KacA, an RHH antitoxin. We here present a phylogenetic analysis of GNAT toxins of more than 1,000 GNAT-RHH pairs detected in completely sequenced K. pneumoniae genomes, revealing that the GNAT toxins are diverse and grouped into four distinct clades. Overexpression of GNAT toxins representative of each of the four clades halts the cell growth of K. pneumoniae, while the coexpression of the cognate RHH antitoxin neutralizes GNAT toxicity. We also identify point mutations that inactivate the GNAT toxins. Moreover, we observe a cross-interaction between GNAT-RHH pairs encoded by different replicons, where a chromosomal toxin (KacT2) can be neutralized by its cognate RHH antitoxin (KacA2 on a chromosome) and another antitoxin (KacA3 on a plasmid). Finally, statistical analysis of the distribution of GNAT-RHH loci in K. pneumoniae strains shows pronounced deviation from random distribution within the same clades. Moreover, we also obtain statistically significant correlations between different clades, which we discuss in terms of the experimental results.

**IMPORTANCE** Elucidating the roles of multifaceted GNAT-RHH TA loci is essential for understanding how these TAs interact among themselves. Recently, the reaction mechanisms and structures of several GNAT-RHH pairs have been reported. While bacterial strains can carry multiple GNAT-RHH loci with diverse origins, studies on the possible cross-interactions of these TA pairs are still limited. Here, we find that 1,000 predicted GNAT toxins of K. pneumoniae can be grouped into four distinct clades. The distributions of TA loci within these clades in K. pneumoniae strains are highly nonrandom, with the presence of a single locus of each clade per strain being highly overrepresented. Moreover, the toxicity of a GNAT toxin encoded by a chromosome was alleviated by a noncognate RHH antitoxin on a plasmid. These results might yield a profound understanding of the widespread GNAT-RHH TA pairs and the cross-interactions between noncognate TA pairs located on different replicons.

## INTRODUCTION

Klebsiella pneumoniae is a nosocomial pathogen that causes many fatal infections (1). Due to its ability to develop resistance to antibiotics, including carbapenems, the World Health Organization (WHO) has categorized it as “critical” in the global priority list for research and development of new antibiotics (2, 3). Also, the pathogenicity of carbapenem-resistant K. pneumoniae strains has evolved, which might lead to its persistence and yield potentially untreatable, recalcitrant infections (4, 5).

Bacterial toxin-antitoxin (TA) modules were first found to be involved in the stable maintenance of plasmids (6), where they interfere with bacterial growth and/or viability when a bacterial cell loses its plasmid (7). TA modules are widespread in bacterial and archaeal genomes (both chromosomes and plasmids) (8). They have been classified into eight types (types I to VIII) based on how the antitoxin neutralizes its cognate toxin (9). A type II TA module consists of a stable toxin protein and an unstable antitoxin protein, which form a nontoxic complex. For example, the Gcn5-related *N*-acetyltransferase (GNAT) toxin is encoded by an operon that also codes for an antitoxin protein that contains a ribbon-helix-helix domain (RHH) (10). To date, eight GNAT toxin proteins of the TA family of GNAT-RHH modules have been characterized, namely, AtaT (11), AtaT2 (12), and ItaT (13) from Escherichia coli, TacT, TacT2, and TacT3 from Salmonella enterica serovar Typhimurium (14–17), GmvT from Shigella flexneri pINV plasmids (18), and KacT from K. pneumoniae (19). In K. pneumoniae, the KacT toxin pairs up with the KacA antitoxin and exists as a GNAT-RHH TA module (19, 20). The KacT toxin was found to halt cell growth and induce antibiotic tolerance (19).

Since TA gene loci are abundant in bacterial strains, it is possible they could interact. Cross-interaction or cross-regulation might occur between cognate and noncognate TA pairs (21–23). For example, a study on the cross-interactions of 20 pairs of ParDE TA modules reveals that ParE toxins’ toxicity can be neutralized by both their cognate ParD antitoxin and the ParD antitoxins of other ParDE TA operons (24). Hence, the TA modules found on the chromosome might also communicate with their counterparts on plasmids. Nevertheless, the latest study on three paralogous TacAT pairs in the S. enterica serovar Typhimurium genome reported that cross-interaction was not observed between the diverse GNAT-RHH pairs (16). The biological relevance of cross-interactions between TA modules is still obscure (23, 25), especially for the various GNAT-RHH modules contained by different bacteria.

We previously reported the distribution of type II TA loci in 10 K. pneumoniae genomes (26) and then characterized one of the GNAT-RHH TA loci, KacAT (19, 20). In this study, we compare diverse GNAT-RHH loci of K. pneumoniae and explore the interactions between chromosomal and plasmid-borne GNAT-RHH pairs. This study increases our understanding of the biological significance and the mechanisms of toxin inactivation in the family of GNAT-RHH TA modules.

## RESULTS

### Distribution and phylogenetic analysis of GNAT-RHH TA loci in K. pneumoniae.

We predicted a total of 7,809 and 3,164 putative type II TA loci in the 499 chromosomes and 2,514 plasmids, respectively, of the completely sequenced K. pneumoniae strains currently available in NCBI RefSeq (Table S1). Similar to our previous results taken from 10 K. pneumoniae genomes (26), RelE or RelE-like toxin is the most represented toxin family in both chromosomes (27%, 2,142/7,809) (Fig. S1A in the supplemental material) and plasmids (27%, 867/3,164) (Fig. S1B). The GNAT-RHH pairs containing a GNAT toxin were ranked as the third most prevalent TA family in the chromosomes (10%, 796/7,809), while they ranked fourth in the plasmids (11%, 359/3,164). Remarkably, 1,155 GNAT-RHH pairs were found in 3,013 K. pneumoniae replicons. All the GNAT toxins found in K. pneumoniae were only paired with the RHH antitoxins. On average, a K. pneumoniae chromosome has two pairs (mean ± standard deviation, 1.60 ± 0.56) of GNAT-RHH loci.

**FIG 1:**
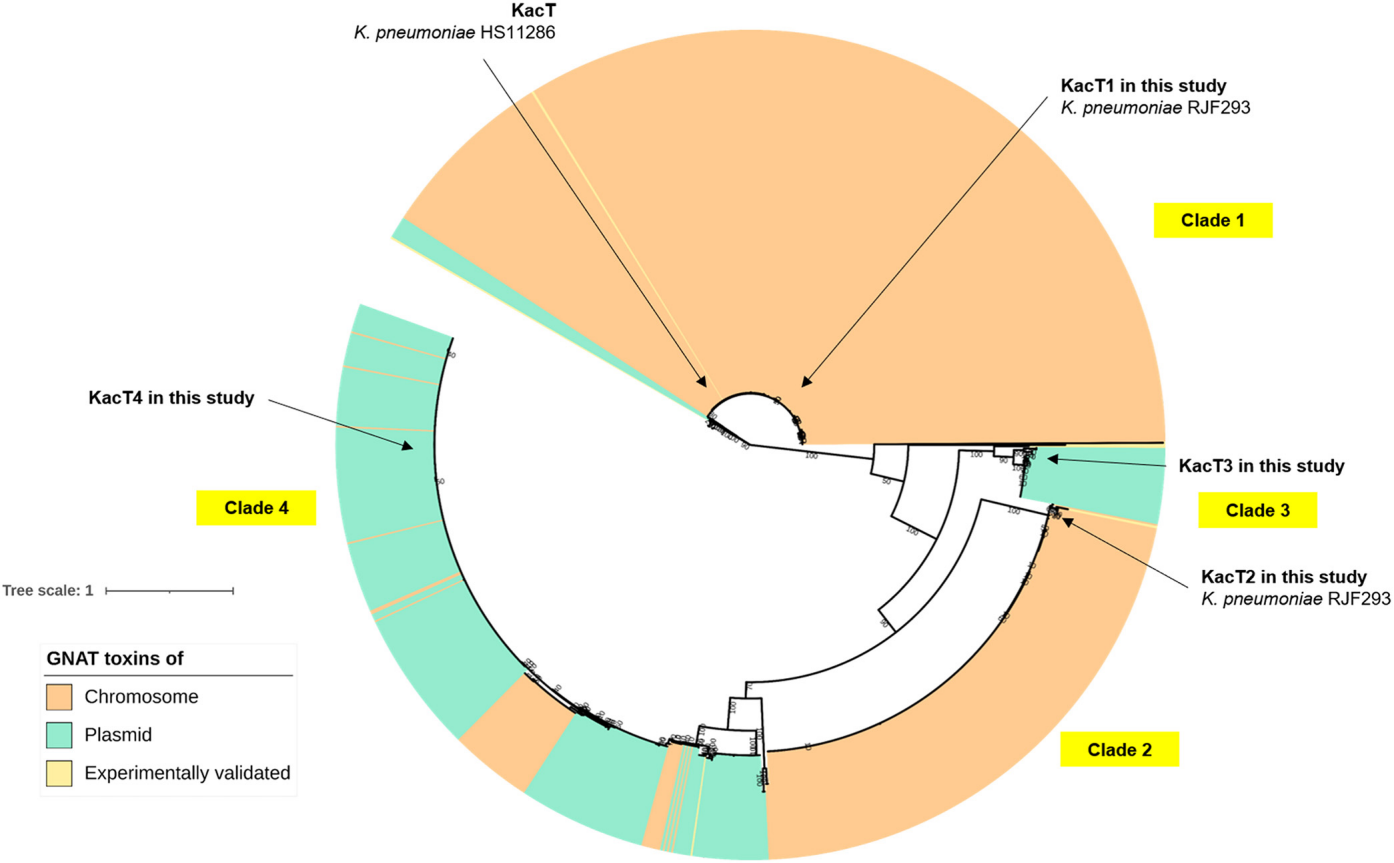
Inferred phylogenetic relationships of the 1,155 acetyltransferase toxin proteins encoded by the GNAT-RHH TA loci in both the chromosomes (*n *= 796) and the plasmids (*n *= 359) of the 499 completely sequenced K. pneumoniae strains. They are grouped into four major clades. Five GNAT toxin proteins of K. pneumoniae are marked with arrows. More details are available in Fig. S2. The maximum-likelihood tree was generated using IQ-TREE (38) with the default settings and displayed using iTOL (39) with midpoint rooting. The details of the K. pneumoniae GNAT toxins are available in Table S2.

After performing a phylogenetic analysis of the 1,155 GNAT toxin protein sequences of K. pneumoniae, four distinct clades were observed (Fig. 1; Fig. S2, Table S2). Notably, the GNAT toxins present on chromosomes were primarily found in clades 1 and 2, while the GNAT toxins present on plasmids were found in clades 3 and 4 (Fig. 1, labeled in green). As many as 499, 250, 36, and 370 GNAT toxins were grouped into clades 1, 2, 3, and 4, respectively.

**FIG 2:**
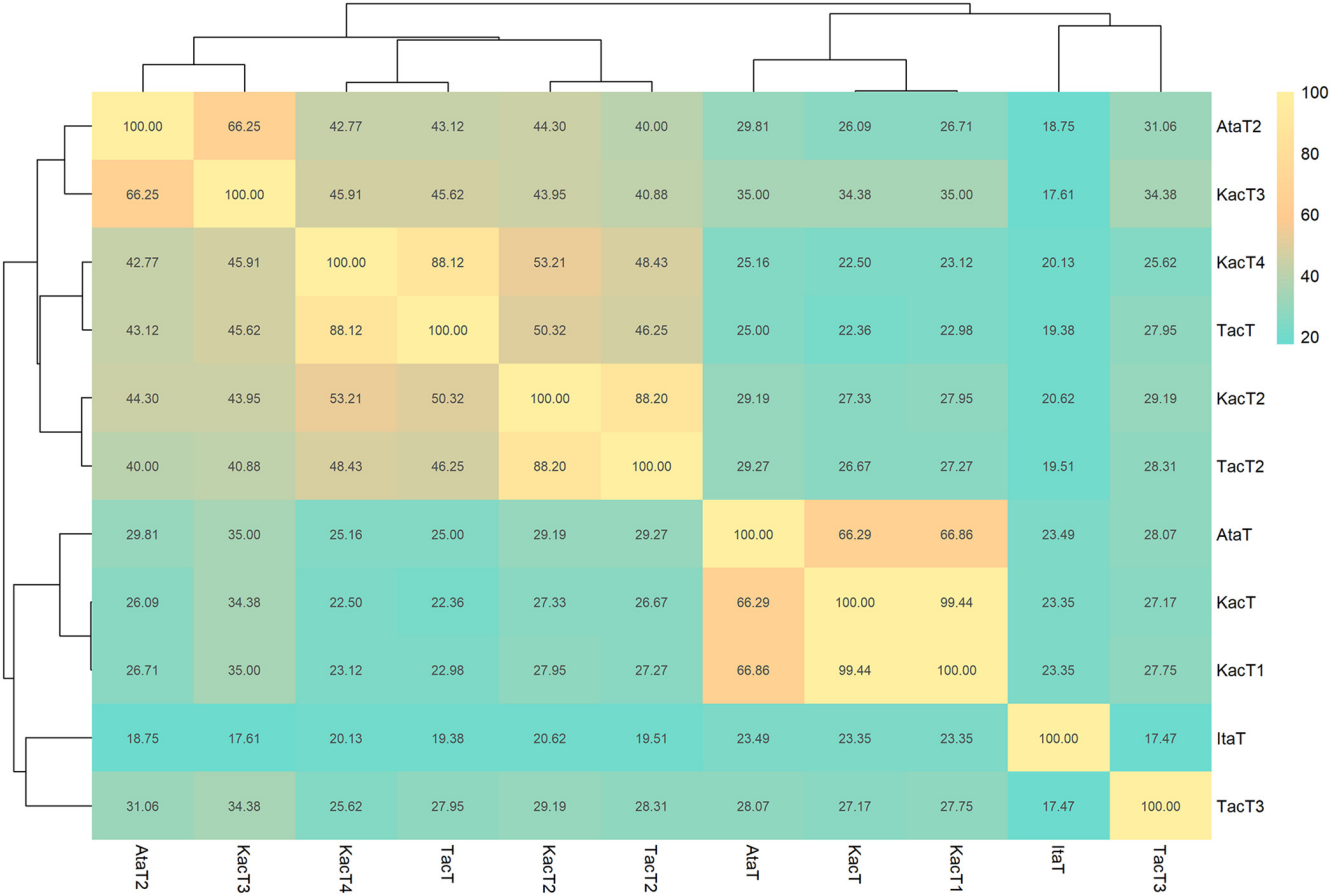
The amino acid sequence identities of GNAT toxins of K. pneumoniae (*n *= 4, KacT1 to -4) compared with those previously reported (*n *= 7) (11–14, 19). Multiple sequence alignments and the percent identity matrix were achieved using Clustal Omega (27). The percent identity matrix was then visualized using the pheatmap (40) function in R. The details of the multiple sequence alignment are available in Fig. S3.

Since the K. pneumoniae GNAT toxins could be classified into four clades, we examined the amino acid sequence similarities among GNAT toxins using Clustal Omega (27). The outcome was presented using the pheatmap function in RStudio (Fig. 2; Fig. S3). We included seven reported GNAT toxins in other species with experimental supports in the comparison. The KacT1 toxin in Clade 1 of K. pneumoniae was somewhat similar to AtaT of E. coli (66.29% BLASTp identity). KacT2 in Clade 2 of K. pneumoniae shared high similarity (88.20% BLASTp identity) with TacT2 of S. enterica serovar Typhimurium. On the other hand, KacT3 and KacT4 were found on K. pneumoniae plasmids. KacT3 in Clade 3 and KacT4 in Clade 4 shared high similarities with AtaT2 on the chromosome of E. coli (66.25% BLASTp identity) and TacT on the chromosome of S. enterica serovar Typhimurium (88.12% BLASTp identity). However, the similarities of the active amino acid residues that were involved in the GNAT-RHH neutralization were low (Fig. S3). In addition, we also examined the coexistence of *kacAT1*, *kacAT2*, *kacAT3*, and *kacAT4* (*kacAT1–4*) TA loci within a single K. pneumoniae strain. Out of 490 K. pneumoniae strains predicted to carry at least a GNAT-RHH TA locus (Table S1), we identified 391 strains carrying multiple copies of the GNAT-RHH TA module within a genome (Fig. S4).

### Bacteriostatic effect of GNAT toxin genes of K. pneumoniae.

**FIG 3:**
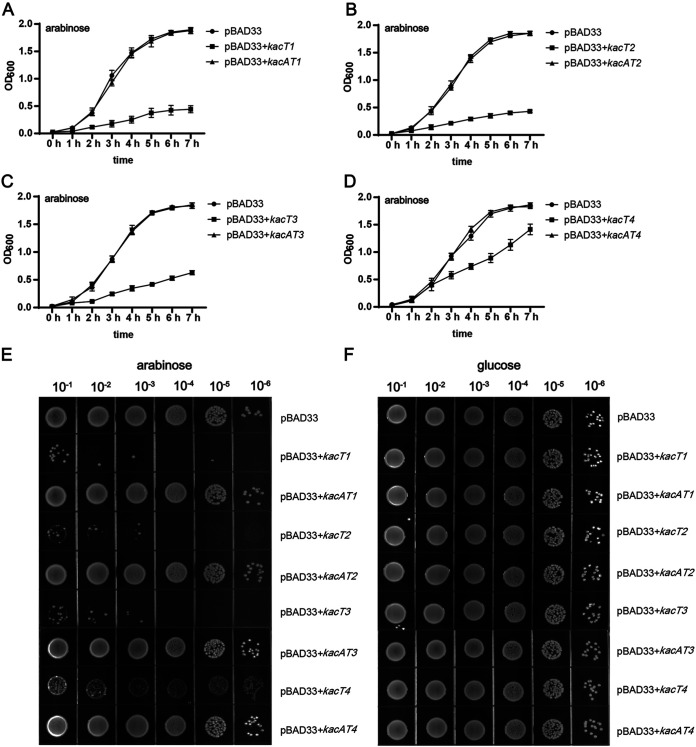
Growth assays performed to assess the toxicities of GNAT toxins of GNAT-RHH TA pairs. (A to D) Growth curves *of* K. pneumoniae HS11286-RR2Δ(*kacAT kacAT2*) with different plasmid combinations. The strains were incubated in LB broth at 37°C. A final concentration of 0.2% arabinose was added to cultures. Optical density at 600 nm was measured at 60-min intervals. Data are presented as the mean values ± standard deviations from three independent experiments. (E and F) Growth of the serially diluted K. pneumoniae HS11286-RR2Δ(*kacAT kacAT2*) with the different plasmid combinations. Strains were plated on LB agar supplemented with 30 μg/mL chloramphenicol complemented with 0.2% arabinose (wt/vol) (E) or 0.2% glucose (wt/vol) (F). The LB plates were cultured overnight at 37°C.

Differences in amino acid sequences were observed among K. pneumoniae GNAT toxins of four different clades. So, it was interesting to learn whether all these toxins were functional. The respective GNAT toxin genes (*kacT1*, *kacT2*, *kacT3*, and *kacT4*) were cloned into the pBAD33 vector and transformed into K. pneumoniae strain HS11286-RR2Δ(*kacAT kacAT2*), a K. pneumoniae HS11286-RR2 mutant strain in which *kacAT* (*kacAT1*) and *kacAT2* are deleted. The effects of GNAT toxin genes on growth were assessed. Briefly, overnight cultures of transformed K. pneumoniae HS11286-RR2Δ(*kacAT kacAT2*) were incubated in LB broth containing chloramphenicol and arabinose. The added arabinose induces transcription of the toxin gene on the pBAD33 plasmid. Growth was monitored for up to 7 h. For the LB agar plate assay, overnight cultures of transformed K. pneumoniae HS11286-RR2Δ(*kacAT kacAT2*) were serially diluted. The dilutions were spotted on the solid LB plates containing chloramphenicol with arabinose or glucose and cultured overnight. We found that the expression of the GNAT toxin genes *kacT1*, *kacT2*, and *kacT3* led to suppression of the growth of K. pneumoniae (Fig. 3) after arabinose induction. KacT4 did not lead to suppressed growth, which will be further discussed below.

In contrast, the expression of GNAT-RHH TA operons (*kacAT1*, *kacAT2*, and *kacAT3*) did not affect the growth of K. pneumoniae HS11286-RR2Δ(*kacAT kacAT2*) (Fig. 3A to D), consistent with the proposal that simultaneous transcription of an antitoxin gene alleviates the bacteriostatic effect of the toxin gene. The pBAD33 plasmid was used to overexpress the toxin gene in this study. The pBAD33 plasmid is an arabinose-inducible expression vector. The pBAD33 plasmid will be overexpressed when arabinose is added into the medium, but not glucose. When the GNAT toxin gene-carrying bacteria were grown in medium supplemented with glucose (where the toxin gene was not expressed), the bacterial growth was not affected (Fig. S5). This phenomenon indicated that growth arrest only occurred when the respective GNAT toxin (KacT1, KacT2, or KacT3) was overproduced under the inducement of arabinose (Fig. 3A to C). We also performed the spot plate assay on LB agar plates. Consistent with the growth curve’s results, the growth of GNAT toxin gene-carrying strains was significantly inhibited (Fig. 3E to F).

### Point mutation of KacT2’s 78th aspartic acid residue abolishes its toxicity.

More than half (58%, 291/499) of the K. pneumoniae strains were found to have two pairs of GNAT-RHH TA loci in their chromosomes. Hence, we next investigated whether these pairs of GNAT-RHH TA loci might be functional. We have previously characterized the first GNAT-RHH locus in the carbapenem-resistant K. pneumoniae strain HS11286 (19, 20). In this study, we examined the KacT2 toxins of both HS11286 (3) and the hypervirulent K. pneumoniae strain RJF293 (28) located in the same clade (clade 2) in the phylogenetic tree (Fig. S2). To differentiate between KacT2s, we named the KacT2 found in HS11286 KacT2_HS11286_ and the KacT2 found in RJF293 KacT2_RJF293_.

**FIG 4:**
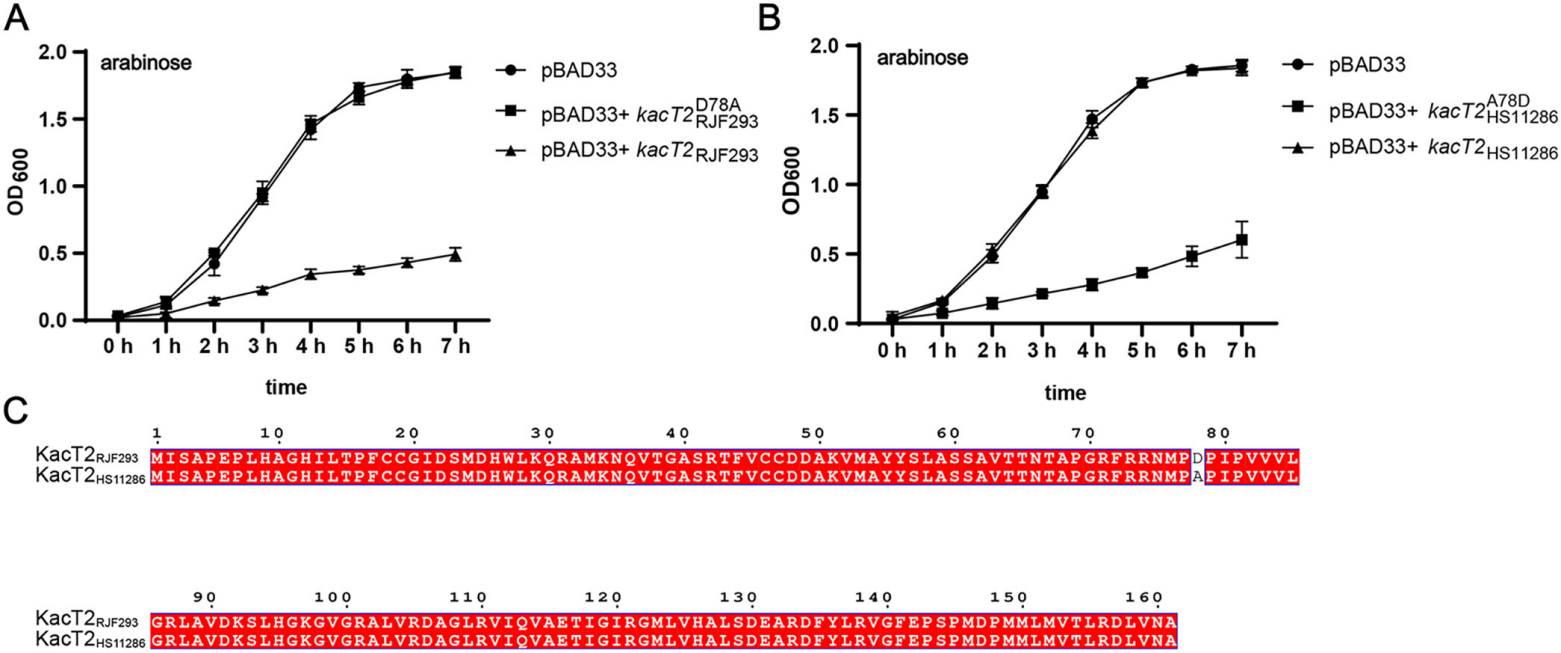
The effects of the amino acid differences between the K. pneumoniae GNAT toxins KacT2_RJF293_ and KacT2_HS11286_ on bacterial growth. (A and B) Growth curves of K. pneumoniae HS11286-RR2Δ(*kacAT kacAT2*) with the plasmid containing the wild-type KacT2_RJF293_, the point-mutated ${\text{KacT2}}_{\text{RJF293}}^{\text{D78A}}$, the wild-type KacT2_HS11286_, or the point-mutated ${\text{KacT2}}_{\text{HS11286}}^{\text{A78D}}$. The bacterial strains were incubated in LB broth at 37°C. Optical density at 600 nm was measured at 60-min intervals. Data are presented as the mean values ± standard deviations from three independent experiments. (C) Amino acid sequence alignment between KacT2_RJF293_ and KacT2_HS11286_.

KacT2_RJF293_ is toxic and suppresses the growth of K. pneumoniae (Fig. 4A). However, we found that KacT2_HS11286_ did not exhibit toxicity (Fig. 4B). The amino acid sequence alignment of KacT2_RJF293_ and KacT2_HS11286_ shows that they differ only in the 78th amino acid residue, which is an aspartic acid residue in KacT2_RJF293_ and an alanine residue in KacT2_HS11286_ (Fig. 4C). This prompted the idea that the nontoxicity of KacT2_HS11286_ was probably due to the amino acid difference. We therefore point mutated the 78th amino acid residue in both KacT2_RJF293_ (from the aspartic acid residue to an alanine residue, ${\text{KacT2}}_{\text{RJF293}}^{\text{D78A}}$) and KacT2_HS11286_ (from the alanine residue to an aspartic acid residue, ${\text{KacT2}}_{\text{HS11286}}^{\text{A78D}}$) to investigate its role in GNAT toxin toxicity. As expected, ${\text{KacT2}}_{\text{RJF293}}^{\text{D78A}}$ had lost its toxicity (Fig. 4A), and the bacteria grew, while ${\text{KacT2}}_{\text{HS11286}}^{\text{A78D}}$ regained toxicity and suppressed bacterial growth (Fig. 4B). Under the glucose condition, these strains containing different plasmids grew normally (Fig. S6). Hence, we concluded that the 78th aspartic acid residue plays a vital role in maintaining the toxicity of KacT2 of K. pneumoniae.

### Nontoxicity of KacT4 is due to its initiation codon.

**FIG 5:**
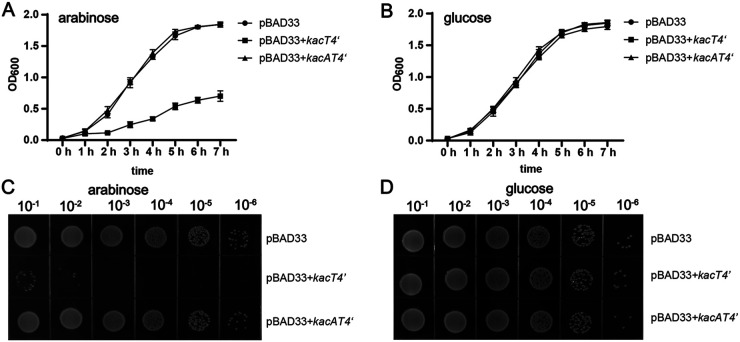
Growth assays to assess the toxicity of the GNAT protein KacT4′. (A and B) Growth curves of K. pneumoniae HS11286-RR2Δ(*kacAT kacAT2*) with different plasmid combinations. The strains were incubated in LB broth at 37°C, supplemented with a final concentration of 0.2% arabinose (A) or 0.2% glucose (B). Optical density at 600 nm was measured at 60-min intervals. Data are presented as the mean values ± standard deviations from three independent experiments. (C and D) Growth of serially diluted K. pneumoniae HS11286-RR2Δ(*kacAT kacAT2*) with different plasmid combinations plated on LB agar with a final concentration of 0.2% arabinose (C) or 0.2% glucose (D). The LB plates were cultured at 37°C overnight.

The GNAT toxin proteins KacT1–3 of K. pneumoniae showed bacteriostatic effects on the host when overexpressed, but the GNAT toxin KacT4 belonging to clade 4 did not (Fig. 3D and E). We repeatedly tested the other two GNAT toxins from clade 4, KacT4α and KacT4β, after arabinose induction, but to no avail (Fig. S7A). As KacT4 shares a very high amino acid sequence identity (Fig. 2; Fig. S3) with the reported GNAT protein TacT of S. enterica serovar Typhimurium, we guessed that it might be functional. The genetic organization of the *kacAT4* locus complies with the classical GNAT-RHH TA locus, with the RHH antitoxin gene (*kacA4*) located upstream from the toxin gene (*kacT4*), as shown in Fig. S8Aiv. The two open reading frames overlap by 14 bp, suggesting translational coupling. After scrutinizing all 370 GNAT gene sequences in clade 4, we realized that 99.73% (369/370) of the KacT4 translation start codons were GUG and not the typical AUG, except for CUG on plasmid pUUH239.2 (Table S3). We subsequently performed heterologous expression assays in K. pneumoniae HS11286-RR2Δ(*kacAT kacAT2*) using an arabinose-inducible promoter with two plasmid-encoded versions of *kacT4*: (i) *kacT4*, with its native initiation codon (GUG), and (ii) *kacT4*′, with the optimized initiation codon (AUG). Using a similar strategy, the functionality of the *kacAT4* and *kacAT4*′ TA loci was also tested. Our results reveal that KacT4′ with an optimized initiation codon (AUG) is required for KacT4 to be expressed at a level that suppresses cell growth of K. pneumoniae (Fig. 5; Fig. S7).

### All *kacAT1–4* TA loci are cotranscribed in an operon.

In a typical type II TA operon, the toxin and antitoxin genes overlap or have a small intergenic region between them. The antitoxin gene is usually located upstream from the toxin gene. As seen in Fig. S8A, the putative GNAT toxin genes *kacT2*, *kacT3*, and *kacT4* overlapped their cognate RHH antitoxin genes, while a 10-bp region separated the GNAT toxin gene *kacT1* and the RHH antitoxin gene *kacA1*. To determine whether all four GNAT-RHH TA loci found in K. pneumoniae were cotranscribed, the total RNA was isolated and reverse transcribed into cDNA. The cDNAs were then amplified using specific oligonucleotide primers (arrows in Fig. S8A and Table S4). We obtained bands with the expected sizes for all four GNAT-RHH TA loci (*kacAT1*, *kacAT2*, *kacAT3*, and *kacAT4*), as shown in Fig. S8B. These results indicated that the *kacAT1–4* gene pairs were cotranscribed as bicistronic operons. The DNA bands for antitoxins (KacA1–4) and toxins (KacT1–4) were detected when the positive-control genomic DNA (gDNA) was used as the template. In contrast, no band was obtained when negative-control total RNA was used as the template (Fig. S8B). In short, our data supported the idea that all four GNAT-RHH TA loci of K. pneumoniae studied (*kacAT1–4*) were organized as the typical type II TA pairs.

### Cross-interaction among four GNAT-RHH TA modules in K. pneumoniae.

Previous reviews suggested that cross-interaction of TA loci might happen among TA modules (21–23). Since a K. pneumoniae genome has an average of 2 pairs of GNAT-RHH TA loci, and since they could be classified into four different clades phylogenetically, we attempted to explore the possibility of cross-interaction among them.

**FIG 6:**
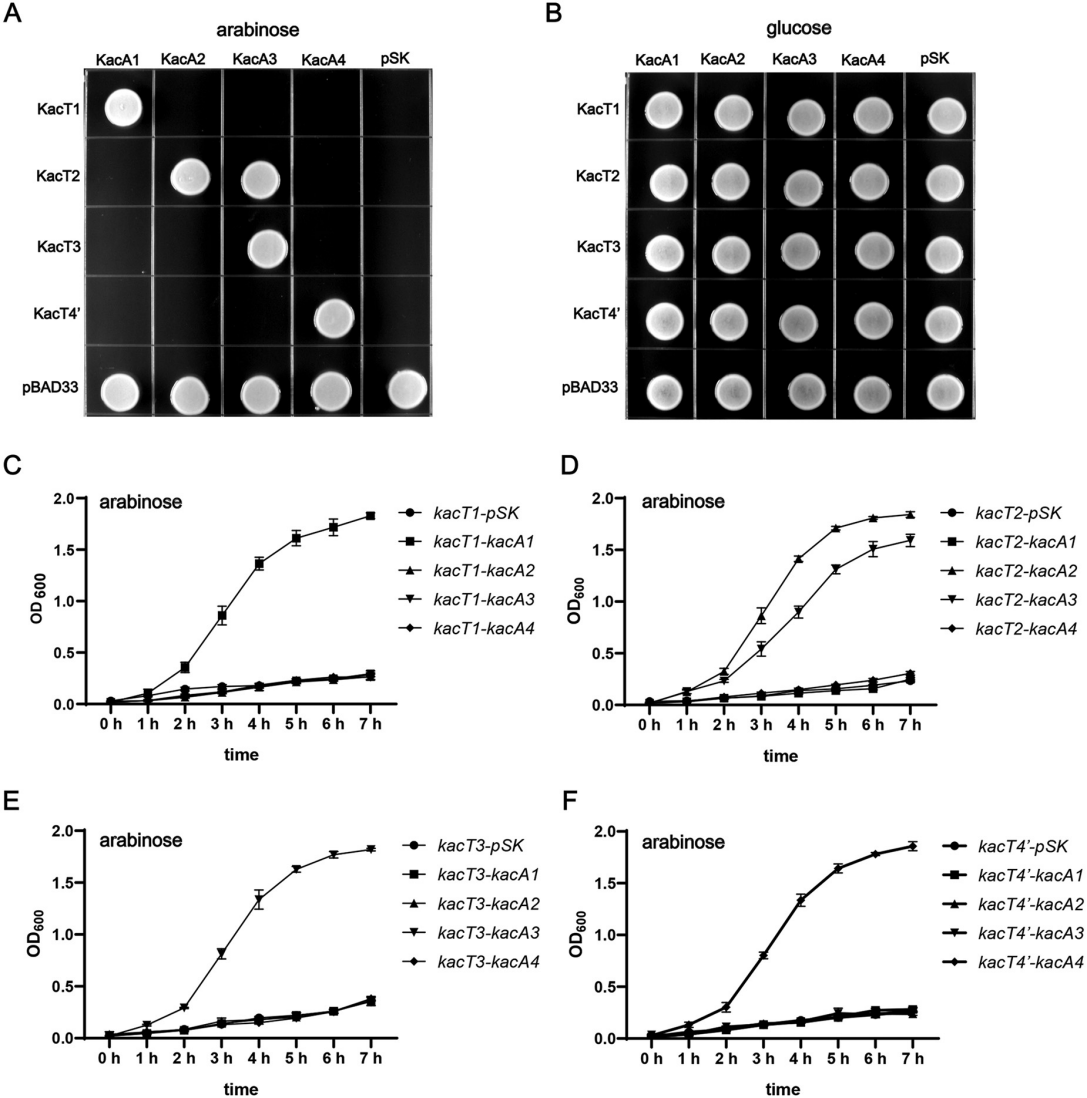
Cross-interactions among different GNAT-RHH TA pairs in K. pneumoniae. (A and B) Growth of K. pneumoniae HS11286-RR2Δ(*kacAT kacAT2*) with different pBAD33-toxin and pSK-antitoxin plasmid combinations plated on LB agar. The LB plates were cultured at 37°C overnight. (C to F) Growth curves of K. pneumoniae HS11286-RR2Δ(*kacAT kacAT2*) strains containing different pBAD33-toxin and pSK-antitoxin plasmid combinations. The strains were incubated in LB broth at 37°C, supplemented with a final concentration of 0.2% arabinose. Optical density at 600 nm was measured at 60-min intervals. Data are presented as the mean values ± standard deviations from three independent experiments. The controls for the growth curve incubated in LB broth supplemented with glucose are available in Fig. S10.

The RHH antitoxin genes (*kacA1–4*) and the GNAT toxin genes (*kacT1–3* and *kacT4*′) were cloned onto the vectors pBluescript SK+ (pSK) and pBAD33, respectively. The pBAD33-toxin and pSK-antitoxin plasmid combinations were then transformed into K. pneumoniae HS11286-RR2Δ(*kacAT kacAT2*). As shown by the results in Fig. 6A, the toxicity of KacT1, KacT3, and KacT4′ could only be neutralized by their cognate antitoxins (KacA1, KacA3, and KacA4). However, interestingly, the toxicity of KacT2 was not only neutralized by its cognate antitoxin KacA2 but was also alleviated by the noncognate KacA3 antitoxin. This result remained consistent when the experiment was repeated with serial dilutions (Fig. S9). We furthermore carried out complementary growth curve assays (Fig. 6C to F; Fig. S10) to study the cross-interaction between GNAT-RHH TA pairs. The results were consistent with those of the experiment whose results are shown in Fig. 6A. Hence, we concluded that there was no cross-interaction between KacAT1, KacAT3, and KacAT4′. Cross-interactions only happened between KacAT2 and KacAT3, where in addition to its cognate KacA2, a noncognate KacA3 antitoxin could alleviate the toxic effect of KacT2.

### Analysis of GNAT-RHH TA locus distribution in K. pneumoniae.

**FIG 7:**
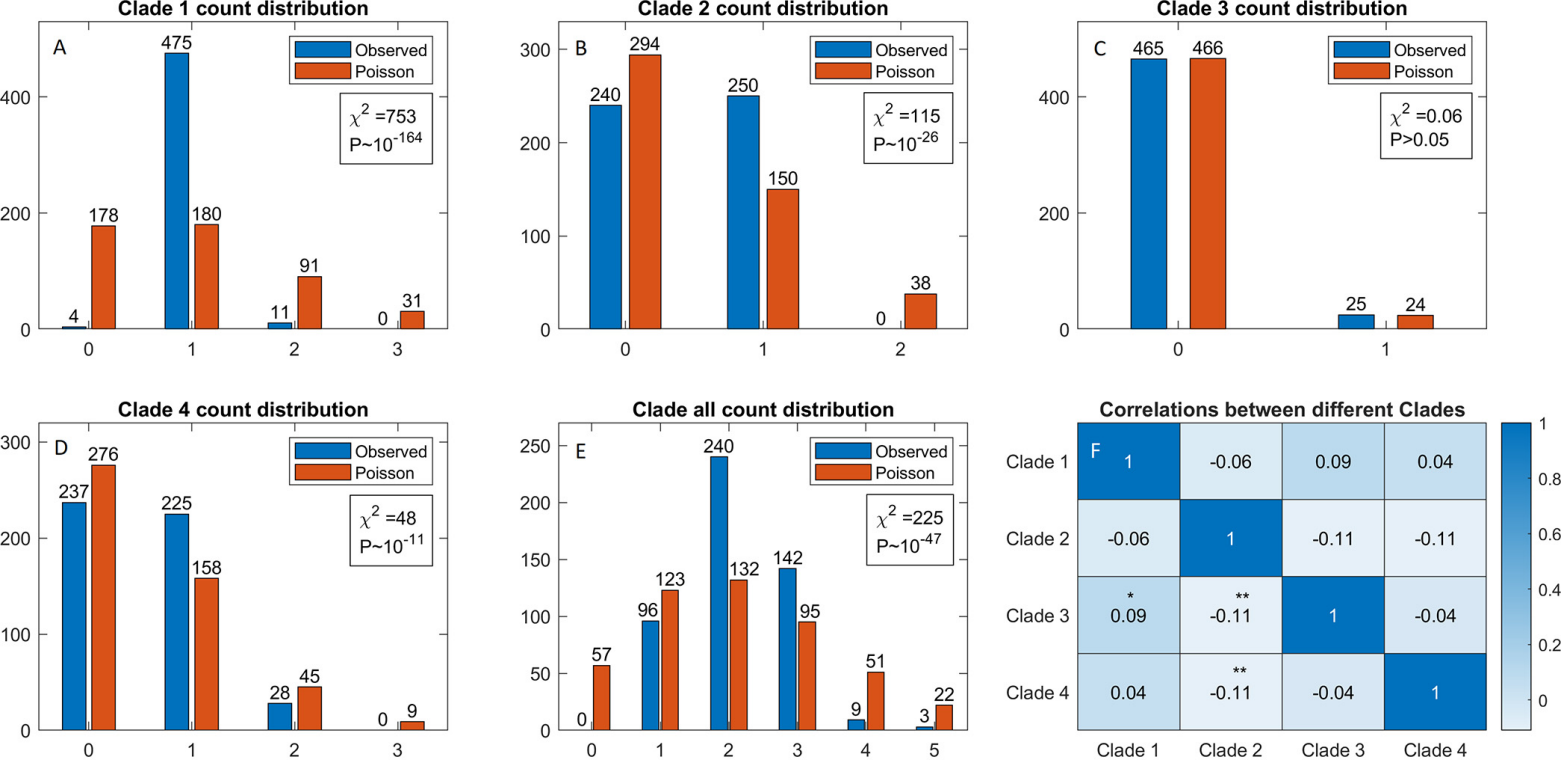
Statistical analysis of count distributions of GNAT-RHH TA pairs of clades 1 to 4 in K. pneumoniae strains. (A to E) Comparison of the observed and the Poisson distribution counts for clades 1 to 4 (A to D) and (E) all classes together. Counts are indicated on the horizontal axis and count frequencies on the vertical axis of bar plots. The exact frequency for each count is indicated above the bar. Differences between the observed and the corresponding Poisson distributions are indicated by χ^2^ and *P* values. (F) Spearman correlation coefficients between the counts of different GNAT-RHH clades in K. pneumoniae strains are shown in the table, with clades indicated along the horizontal and vertical table dimensions. A color key indicating the magnitude of correlations is shown on the right of the table. The statistically significant correlations are indicated by asterisks as follows: **, *P* value of ~0.01; *, *P* value of ~0.1.

We next analyzed the distribution of GNAT-RHH TA loci belonging to different clades. We wanted to assess (i) whether the distribution of TA loci within a given clade departed from randomness, i.e., whether these loci tended to cluster together, and (ii) whether the counts of TA loci belonging to different clades were mutually correlated, which would indicate interdependences of these clades. In Table S5, we assembled the counts for each clade (table columns) for each sequenced strain (table rows) to address the questions above. To account for the possibility of interactions between loci on a chromosome and a plasmid, the counts included loci on both the chromosome and plasmids for a given strain. Next, we fitted a Poisson distribution to the counts in the table, corresponding to the null hypothesis that TA loci were distributed independently and at the same rate among the strains. The mean values of the Poisson distributions were inferred independently for counts in each clade and for counts in all four clades together. Comparisons between the Poisson and the actual (observed) distributions are shown for individual clades in Fig. 7A to D.

To assess statistical significance, i.e., the null hypothesis that counts were randomly distributed, we used the χ^2^ test to compare the differences between the observed and the Poisson distributions. The χ^2^ values and statistical significance values (*P* values) obtained for each clade are indicated in the corresponding panels in Fig. 7. The most significant deviation was obtained for clade 1 (Fig. 7A), where the presence of only one locus was strongly overrepresented, while both the absence and the presence of more than one locus was strongly disfavored. Though somewhat less pronounced, the same pattern was also observed for clades 2 and 4, where we also found statistically highly significant deviations from the Poisson distributions (Fig. 7B and D). Such deviations were not a generic property of TA loci, as the clade 3 counts closely followed the Poisson distribution (Fig. 7C).

Finally, for all GNAT-RHH TA loci (i.e., all clades together), two or three instances were favored, while more than three loci or the absence of loci was disfavored (Fig. 7E; Fig. S4), with statistically highly significant deviations from the Poisson distributions. This was consistent with the strong overrepresentation of one locus for clades 1, 2, and 4.

Correlations between GNAT-RHH locus counts in sequenced K. pneumoniae strains are shown in Fig. 7F. Spearman correlations, with the corresponding significance levels, are shown, to take into account that the counts do not follow the normal distribution and for possible nonlinear relations. However, both the Pearson and the Kendall correlations led to the same conclusion. We found significant (*P* = 0.01) negative correlations between clade 2 and clades 3 and 4. The magnitudes of these correlations were not high, but they were robustly obtained as statistically significant for all three correlation methods. The result was interesting, as the interactions among clades 2 and 3 were experimentally observed. These negative correlations were not a generic consequence of clade 2 being on the chromosome and clades 3 and 4 on plasmids, since the correlation of clade 1 (also on the chromosome) and clade 3 was positive (marginally statistically significant, *P* = 0.05).

## DISCUSSION

In this study, we performed an *in silico* search of bacterial type II TA loci using TAfinder (29) and identified a total of 1,155 GNAT-RHH TA loci among 3,013 publicly available K. pneumoniae replicons. The diverse GNAT toxins of K. pneumoniae could be phylogenetically grouped into four clades. Although the GNAT toxins’ amino acid similarities between different clades were low, they were functional and represented characteristics similar to those of other classical type II TA modules. All of the GNAT toxins inhibited bacterial growth when overexpressed, and their cognate antitoxins could neutralize the toxic effect. In addition, all of the GNAT-RHH TA gene pairs were transcribed in a bicistronic operon. Moreover, we observed a cross-interaction between a chromosomal GNAT toxin protein (KacT2) and a plasmid-borne RHH antitoxin protein (KacA3) where the KacA3 antitoxin could alleviate KacT2’s toxicity.

On average, a K. pneumoniae strain carried two pairs of GNAT-RHH TA loci. The GNAT-RHH TA loci were found to regulate bacterial cell growth (11–19). In K. pneumoniae, the GNAT toxin (KacT) inhibits cell growth and, during overproduction, induces antibiotic tolerance (19). In this study, we phylogenetically classified the 1,155 putative GNAT toxins into four clades, with clades 1 and 2 predominantly containing toxins encoded by the chromosomes, while clades 3 (KacT3) and 4 (KacT4) were those mainly encoded by plasmids. We found that GNAT toxins across the clades were similar to some GNAT toxins of K. pneumoniae that were previously discovered in other species: clade 1 was similar to KacT (19) and AtaT (11) of E. coli, clade 2 was similar to TacT2 of S. enterica (15, 16), clade 3 was similar to AtaT2 of E. coli (12), and clade 4 was similar to TacT1 of S. enterica (14) (Fig. 2; Fig. S3). However, the similarities across the toxin-antitoxin neutralization domain were low (Fig. S3). In the chromosome of E. coli, a second pair of a GNAT-RHH TA locus, *ataRT2*, was also observed and studied (12); AtaT2 shares 66.25% BLASTp identity with KacT3 that was identified on a plasmid of K. pneumoniae. Only ItaT of E. coli showed low similarities (less than 23.49% BLASTp identities) to all K. pneumoniae GNAT toxins (Fig. 2; Fig. S3). ItaT also developed a distinct subbranch in the phylogenetic trees (Fig. 1; Fig. S2).

Apart from that, despite diverse sequence identities among K. pneumoniae GNAT toxins across different phylogenetic clades, all of them were functional. Our experiments demonstrated that all *kacAT1–4* TA pairs of K. pneumoniae exhibited typical type II TA module features. They were cotranscribed simultaneously and had bacteriostatic effects when the toxins (KacT1–4) were overproduced (19).

We also discovered that a single amino acid change could affect the toxicity of GNAT toxins. Here, we present two scenarios where a GNAT toxin’s toxicity was attenuated. First, in KacT2, we discovered that the toxin’s toxicity was affected by the 78th amino acid residue. Instead of an alanine residue, a toxic KacT2 should have an aspartic acid residue as its 78th amino acid residue. This phenomenon suggested that aspartic acid might function as an active-site residue involved in substrate catalysis. However, it is noteworthy that a dimeric state of the GNAT toxin is essential for acetyltransferase activity (14, 19, 30). Thus, the 78th aspartic acid residue might also be involved in maintaining the dimerization of KacT complexes.

Aside from KacT2 of K. pneumoniae, we also observed another incident of attenuated toxicity for KacT4s. The point mutation of GUG to AUG showed that the malfunction of KacT4s was due to a point mutation at the initiation codon. A similar phenomenon where the toxin’s toxicity was affected by the initiation codon was also previously observed in RES-Xre TA modules (31). GUG is an ambiguous code and a noncanonical start codon (32). In annotated bacterial genomes, AUG is the most common start codon (81.80%), followed by GUG (13.80%) (32). When AUG is absent, GUG acts as a start codon by encoding a methionine residue. Although only a single nucleotide base difference was observed in position 1 of the start codons, the peptide synthesis might be affected. However, we suggest that the mutation will not affect peptide synthesis in our case because bacteria can mutate the Shine-Dalgarno sequence to compensate for the suboptimal start codon (GUG), thus increasing the translation initiation signal (33). One possible explanation for the change in KacT4’s toxicity is that the point mutation for the start codon happens over evolutionary time scales, resulting in a less toxic KacT4 (32).

This study also examined the interactions between GNAT toxin and RHH antitoxin proteins. Previous studies have suggested that TA modules are likely to cross-regulate among themselves due to their abundance in bacterial genomes (21–23). In this study, we observed a cross-interaction between noncognate GNAT TA pairs of K. pneumoniae. We observed that KacA3 could neutralize the toxicity of its cognate toxin KacT3 and alleviate KacT2’s toxicity, even though the amino acid identities between KacA2 and KacA3 were low (34.44%) (Fig. S11). The *kacAT2* TA locus was predominantly found on chromosomes, while the *kacAT3* TA locus was only on the plasmids. Also, seven K. pneumoniae strains were found to carry *kacAT2* and *kacAT3* at the same time (Fig. S4). This phenomenon suggests that cross-communication among native and acquired TA loci is possible, probably contributing to a more extensive cellular regulatory network.

There are few reports about cross-interaction among noncognate TA pairs of the same family, even though they are located within the same chromosome (34). Most recently, Grabe et al. reported no cross-interactions among three GNAT-RHH TA modules in S. enterica serovar Typhimurium (TacAT, TacAT2, and TacAT3), where the TacA antitoxins only specifically neutralize their cognate TacT toxins (16). The non–cross-interaction is due to the diverged α4 helix on the TacT toxin, acting as the auxiliary interface and driving the specific recognition of its cognate antitoxin (16). However, some contradicting findings suggest that TA cross-interaction can occur between members of the same TA family from different bacterial species, such as ParDE pairs (24). We also observe that RelB antitoxin of E. coli, RelBeco, alleviated the toxicity of RelE toxin RelE2sca of Streptomyces cattleya (35).

Apart from that, cross-interaction was also observed within members of the same TA family but of different replicons, that is, on chromosome and plasmid, respectively (21, 22). For instance, in Erwinia chrysanthemi, a chromosomally encoded *ccdEch* system can interfere with *ccdF*, a close homologue of *ccdE*, on the plasmid (36). This situation was described as antiaddiction: the chromosomal TA system will express its antitoxin to neutralize the invading toxin to compensate for the loss of a TA-carrying-plasmid, preventing postsegregational killing caused by TA modules (21–23, 36). Notably, we observed the toxicity of a chromosomal toxin (KacT2) to be alleviated by a plasmid-borne antitoxin (KacA3) in an E. coli model, which is slightly different from the established antiaddiction model. Besides, we speculate that KacT2 and KacT3 toxins have similar auxiliary interfaces that can recognize the KacA3 antitoxin (16), allowing cross-interaction. Thus, further investigations are warranted to elucidate the interactions and toxin-antitoxin specificities between different GNAT-RHH TA pairs in K. pneumoniae.

The interactions between GNAT-RHH pairs discussed above led us to investigate how GNAT-RHH TA loci were distributed among K. pneumoniae strains. For clades 1, 2, and 4, we found that the distribution of TA loci counts deviated significantly from randomness, where the presence of one locus per strain was strongly overrepresented at the expense of either the absence of loci or the presence of multiple loci. The presence of at least one locus may be strongly preferred due to the functional importance of clades 1, 2, and 4 in K. pneumoniae strains. On the other hand, the presence of multiple loci within the same clade may be functionally redundant or lead to undesired cross talk between TA pairs. It is interesting to note that the presence of multiple copies of similar TA loci has been proposed to increase cooperativity in their dynamics and promote bistability and the formation of persister cells (37). However, such TA system multiplicity appears to be disfavored, at least for GNAT-RHH pairs.

On the other hand, associations of GNAT TA loci of different clades appeared to be weak, consistent with the absence of experimentally observed interactions between most clades. However, in line with observed interactions between clades 2 and 3, we found statistically significant correlations between the count numbers of these two clades. Negative correlations indicated that some of the interactions between the two clades might be disfavored, perhaps because they would lead to undesired cross talk between the GNAT-RHH pairs. Still, a statistically significant negative correlation was also found between clades 2 and 4, where interactions were not experimentally observed. Consequently, the negative association may also be due to reasons other than direct physical interactions, e.g., in some instances, the presence of clade 2 might make the presence of clade 4 functionally redundant (and vice versa). Such possibilities need to be experimentally investigated in the future.

Overall, this study investigated the distribution and characteristics of GNAT-RHH TA loci in K. pneumoniae. This study also compared four GNAT-RHH pairs of different clades and observed a cross-interaction between chromosomal and plasmid-borne GNAT-RHH TA pairs.

## MATERIALS AND METHODS

### Genome sequences and annotations.

A total of 3,013 complete nucleotide sequences and annotations of K. pneumoniae genomes (499 chromosomes and 2,514 plasmids) were retrieved from the NCBI RefSeq database in May 2020. Only complete genomes were included in this analysis to ensure the quality of the output.

### Bioinformatics analysis.

The type II TA loci were predicted using TAfinder with the default settings (29), utilizing the Toxin-Antitoxin Database (TADB 2.0). It predicts type II TA pairs in bacterial genomes via homology searches and operon structure detection (29). To perform phylogenetic analyses, selected amino acid sequences of the GNAT toxin proteins were retrieved from the TAfinder output. Multiple sequence alignment of these GNAT toxin protein sequences was performed using Clustal Omega (27). Then, phylogenetic analysis was carried out via the maximum-likelihood approach using IQ-TREE (38). The output was visualized using Interactive Tree of Life (iTOL) with midpoint rooting (39). The percent identity matrix was created using Clustal Omega and visualized using the pheatmap (40) function in R (41).

### Bacterial strains, plasmids, and growth conditions.

Klebsiella pneumoniae strain HS11286-RR2Δ(*kacAT kacAT2*), K. pneumoniae strain RJF293, plasmid pBAD33, and pBluescript SK+ (pSK) were used in this study. K. pneumoniae HS11286-RR2Δ(*kacAT kacAT2*) is a strain in which the *kacAT* (*kacAT1*) and *kacAT2* genes are deleted. In the K. pneumoniae HS11286 mutant HS11286-RR2, the plasmid-carrying *bla*_KPC-2_ and 26-kb multiple-drug-resistance regions have been deleted, which allows a flexible use of antibiotic selection markers. All primers were synthesized by Genewiz (Suzhou, China). The details of bacterial strains and plasmids (including primer sequences) are listed in Table S4. The pBAD33-carrying K. pneumoniae HS11286-RR2Δ(*kacAT kacAT2*) cells were cultivated either in Luria-Bertani (LB) broth or on LB agar plates at 37°C, supplemented with chloramphenicol (30 μg/mL). In addition, pBAD33 contains an arabinose-induced promoter, *araBAD*. Hence, arabinose (0.2%, wt/vol) was added to the culture medium to induce *araBAD* expression, while glucose (0.2%, wt/vol) was added to inhibit it.

### Plasmid construction.

To construct plasmids for phenotypic assays in K. pneumoniae HS11286-RR2Δ(*kacAT kacAT2*), the single GNAT toxin genes (*kacT1*, *kacT2*, *kacT3*, and *kacT4*) and their complete GNAT-RHH TA gene operons (*kacAT1*, *kacAT2*, *kacAT3*, and *kacAT4*) were each cloned into pBAD33.

Briefly, the toxin genes and TA operons, including the native ribosomal binding site, were amplified from the genomic DNA (gDNA) of K. pneumoniae RJF293 (28) and synthetic genes. In this study, *kacAT1* and *kacAT2* were amplified from K. pneumoniae RJF293, while *kacAT3* and *kacAT4* were synthesized based on the TA loci found in plasmid pSWHvKp (NCBI RefSeq accession no. NZ_CP054064) and plasmid pKCTC2242 (NC_017541) (42), respectively. The TA operons (HQ868_25586 to HQ868_25590 for *kacAT3* and KPN2242_RS26500 to KPN2242_RS26505 for *kacAT4*) with their respective upstream promoter regions were synthesized.

The corresponding primers used are detailed in Table S4. The PCR products were digested with SacI-HindIII enzymes and cloned into pBAD33, resulting in the plasmids pBAD33+*kacT1*, pBAD33+*kacAT1*, pBAD33+*kacT2*, pBAD33+*kacAT2*, pBAD33+*kacT3*, pBAD33+*kacAT3*, pBAD33+*kacT4*, and pBAD33+*kacAT4*. The plasmids were then transformed into K. pneumoniae HS11286-RR2Δ(*kacAT kacAT2*) using calcium chloride for growth assays.

For the plasmids used to examine cross-interaction among different GNAT-RHH TA loci in K. pneumoniae, the RHH antitoxin genes (*kacA1–4*) were also cloned into pBluescript SK+ (pSK).

### Point mutation.

Point mutations for KacT2 (at the 78th amino acid residue) and KacT4 (at the initiation codon) were achieved using the QuickMutation site-directed mutagenesis kit (catalog no. D0206) from Beyotime Biotechnology (Shanghai, China). We performed the point mutations following the manufacturer’s instructions.

### Growth assay.

Overnight cultures of transformed K. pneumoniae HS11286-RR2Δ(*kacAT kacAT2*) were diluted 1:100 into fresh LB broth supplemented with 30 μg/mL of chloramphenicol and 0.2% arabinose (wt/vol). The arabinose was added to the broth before the culture. Then, the culture was incubated at 37°C. Growth was monitored by determining the optical density at 600 nm (OD_600_) at 60-min intervals up to 7 h. For the LB agar plate assay, overnight cultures of transformed K. pneumoniae HS11286-RR2Δ(*kacAT kacAT2*) were serially diluted. The dilutions were spotted (10 μL) on the solid LB plates containing 30 μg/mL chloramphenicol and 0.2% arabinose (wt/vol) or 0.2% glucose (wt/vol). The LB plates were cultured at 37°C overnight (19, 43).

### Cotranscription assay.

A cotranscription assay was performed to investigate whether the GNAT toxin gene and RHH antitoxin gene pair could be cotranscribed. Total RNA was extracted using the RNeasy kit (Qiagen, Germany) according to the manufacturer’s instructions. After digesting the gDNA using DNase I, 500 ng RNA was converted to cDNA using the PrimeScript reverse transcription (RT) reagent kit (TaKaRa, Japan). Different primers (shown in Fig. S8A) were designed for PCR amplification using cDNA, RNA, and gDNA templates. The PCR products were then examined by agarose electrophoresis.

For the cotranscription assay of synthetic *kacAT3* and *kacAT4*, we cloned the synthetic genes (with their upstream promoter regions) into the pBAD33 vector and transformed them into K. pneumoniae HS11286-RR2Δ(*kacAT kacAT2*). The transformed strains were cultured in LB medium containing 0.2% arabinose until their growth reached the mid-log phase. Then, the total RNA was extracted and reverse transcribed into cDNA, and PCR was performed using the corresponding primers. The PCR products were then examined by agarose electrophoresis.

### Cross-interaction assay.

We interrogated the potential cross-interactions among the four GNAT-RHH pairs in K. pneumoniae. We cloned the GNAT toxin gene and the RHH antitoxin gene onto pBAD33 and pBluescript SK+ (pSK), respectively, using restriction-based cloning. The plasmids have different transcription levels under normal conditions. The primer used for cloning is listed in Table S4. First, we mixed the plasmids containing cognate and noncognate TA combinations. Next, we cotransformed the plasmid combinations into K. pneumoniae HS11286-RR2Δ(*kacAT kacAT2*) as shown in Fig. 6.

### Analysis of GNAT-RHHTA locus distribution.

The Poisson distribution was fitted to the observed GNAT-RHH TA locus counts in K. pneumoniae strains, individually for each clade and for all clades together. The statistical significance of the differences between the Poisson distribution and the observed counts’ distributions was estimated by the χ^2^ test. Spearman, Pearson, and Kendall correlation coefficients and their corresponding *P* values were calculated for counts that belonged to different clades.

### Data availability.

The TAfinder-predicted type II toxin-antitoxin (TA) loci of the 499 completely sequenced K. pneumoniae genomes used are provided in Tables S1 and S2.
