# Supplementary material for: Comparative Analysis of Diverse Acetyltransferase-Type Toxin-Antitoxin Loci in Klebsiella pneumoniae
Source: Microbiol Spectr. 2022 Jun 15;10(4):e00320-22. doi: 10.1128/spectrum.00320-22 (PMC9431474; doi:10.1128/spectrum.00320-22)
Supplement: Supplemental file 1 — Captions to Tables S1 to S5; Fig. S1 to S11. Download spectrum.00320-22-s0001.pdf, PDF file, 2.1 MB [file spectrum.00320-22-s0001.pdf]

SUPPLEMENTARY DATA

Table S1. TAfinder-predicted type II toxin-antitoxin (TA) loci of 501 completely sequenced *K. pneumoniae* chromosome and plasmid genomes. The “Unknown” column consists of underexplored TA family candidates predicted via conserved domains by TAfinder; these TA families are yet to be verified and classified.

Table S2. Details of the TAfinder-predicted GNAT toxin genes in *K. pneumoniae* genomes as illustrated in the phylogenetic trees (**Fig. 1** and **Fig. S2**).

Table S3. Details of KacT4s’ start codon.

Table S4. Oligonucleotides used in this study.

Table S5. GNAT-RHH TA of different clades.

Figure S1. Families of putative toxins coded by type II TA loci in the chromosomes and the plasmids of the 499 completely sequenced *K. pneumoniae* strains.

Figure S2. Inferred phylogenetic relationships of the 1,155 GNAT toxin proteins encoded by GNAT-RHH TA loci of *K. pneumoniae.*

Figure S3. Multiple sequence alignment of GNAT toxins (*n* = 11) with their respective amino acid residues that were involved in toxin-antitoxin neutralization.

Figure S4. Venn diagram showing the existence of multiple *kacAT* TA loci of different clades in a K. pneumoniae strain.

Figure S5. Growth curves of *K. pneumoniae* HS11286-RR2Δ(*kacATkacAT2)* carrying different pBAD33 combinations, induced with glucose.

Figure S6. The effect of the amino acid difference between the *K. pneumoniae* GNAT toxins $KacT2_{RJF293}$ and $KacT2_{HS11286}$ on bacterial growth.

Figure S7. Growth assays to assess the toxicity of the GNAT *protein* KacT4’.

Figure S8. Genetic organization and co-transcription of GNAT-RHH TA operon.

Figure S9. Cross-interaction among different GNAT-RHH TA modules in *K. pneumoniae*.

Figure S10. Growth curve of *K. pneumoniae* HS11286-RR2Δ(*kacATkacAT2)* strains containing different pBAD33-toxin and pSK-antitoxin combinations.

Figure S11. Multiple sequence alignment of amino acid sequence between various RHH antitoxin proteins of *K. pneumoniae*.

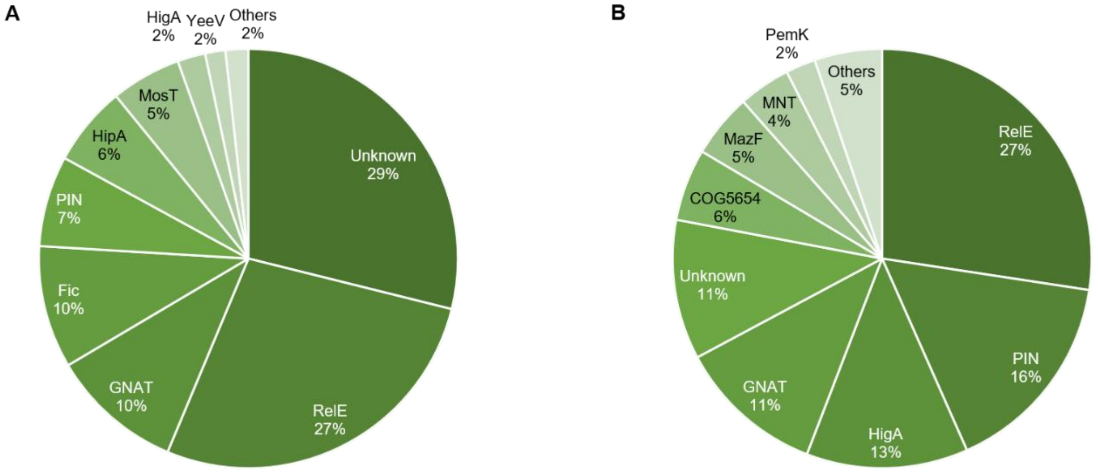

Figure S1. Families of putative toxins coded by type II TA loci in the **(A)** chromosomes and the **(B)** plasmids of the 499 completely sequenced *K. pneumoniae* strains. "Other" consists of under-represented conserved domains, while "Unknown" consists of underexplored TA family candidates predicted via conserved domains by TAfinder; these TA families are yet to be verified and classified. A detailed list of toxins and antitoxins for both chromosomes and plasmid are available in **Table S1**.

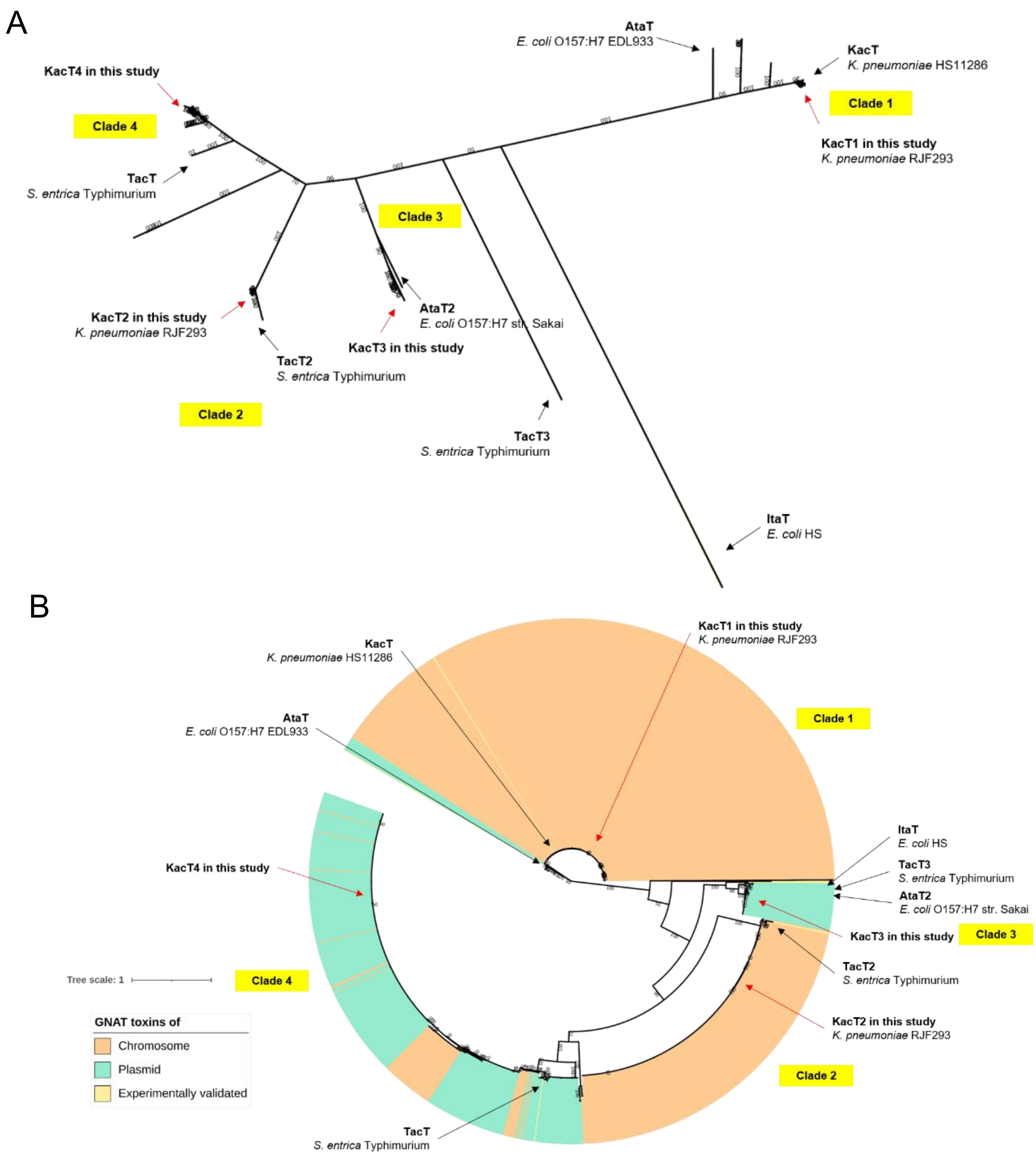

Figure S2. Inferred phylogenetic relationships of the 1,155 GNAT toxin proteins from both chromosomes (*n* = 796) and plasmids (*n* = 359) of *K. pneumoniae*, including 7 GNAT toxin proteins of other species that had been experimentally validated (labeled in black arrows). The toxin proteins that are under analysis in this study are labeled with red arrows. The Maximum Likelihood tree was generated with IQ-TREE with the default settings (1) and displayed by iTOL with **(A)** unrooting and **(B)** midpoint rooting (2).

```
TacT1     ----MGRVTAPEPL-------SAFHQVAEFVSGEAVLDDWLKOKGLKNQALGAARTFVVC    49
TacT2     -------MISTPEPL-------HAGHILTPFCCGVDSIDNWLEQRAMKNQTTGASRTFVCC   47
TacT3     ---MMFTDWHEAAI-------GKTHNRMNFDCGDADLNQFLQRHARQNHEKGTTKTYVAL    50
AtaT      -----MDDLTIEIL-----TDDADYDLQRFDCGEEALNLFLTTHLVRQHRNKILRAYILC    50
AtaT2     -----MGITAPTPL-------TSEHNLADFCCSDHGMNEWLKKKALKNHSSGLSRVYVIC    48
ItaT      MVDKHEEITLPIVLSCNYQSDITYPGQKQFDCGNPVIDKFVRASLKKSVRNSDCAAKALI    60
KacT      ----MEQQLTIEMI-----ADAFSYDITGFDCGEEALNTFLKEHLKRQHDGQILRGYALV    51
KacT1     ----MEQQLTIEMI-----ADAFSYDITGFDCGEEALNTFLKEHLKRQHDGQILRGYALV    51
KacT2     -------MISAPEPL-------HAGHILTPFCCGIDSMDHWLKQRAMKNQVTGASRTFVCC   47
KacT3     -----MGMRAPESL-------MSEHNIAEFCCQEPALNEWLKKKALRNHSTGISRVYVVC    48
KacT4     ----MGRITAPEPL-------SSAHQLAEFVSGETVLDEWLKQRGLKNQALGAARTFVVC    49
                       :               *  .    :: ::     :.

TacT1     K-KDTKOVAGFYSLATGSVNHTEATGNL-RRNMP-DPIPVIILARLAVDLSFHGKGLGAD    106
TacT2     G-SDS-NVLAYYSLASSAVTTNTSPGRF-RRNMP-DPIPVVVLGRLAVDKSLHGQGVARA    103
TacT3     DNSDVTRIHGFYSVSPASLIYAQVPGAI-SKGLGRYDVPVFRLGRLAVDKSMQGQGLGAQ    109
AtaT      RNTPERQVLGYYTLCGSCFERAALPSKSKQKKIPYKNIPSVTLGRLAIDRSLQGQGWGAT    110
AtaT2     I-ANTRQVIGYYCLSTGSIQRNLAPGAM-RRNAP-ESLPVVVLGRLAIDQAWAGKGLGVA    105
ItaT      D-RQSGELIGICTFTAYSLEKQRVSGVL-QGSQP-SEIGVVRLVMLGVARKYQKRGFGQD    117
KacT      SGDTVPRLLGYYTLSGSCFERGMLPSKTQQKKIPYQNAPSVTLGRLAIDKSVQGQGWGEM    111
KacT1     SGDTVPRLLGYYTLSGSCFERGMLPSKTQQKKIPYQNAPSVTLGRLAIDKSVQGQGWGEM    111
KacT2     D--DA-KVMAYYSLASSAVTTNTAPGRF-RRNMP-DPIPVVVLGRLAVDKSLHGKGVGRA    102
KacT3     A-ENTNRVIGYYCLSSGSVHRNTVPGAY-RRNAP-DVIPVIVLGRLAIDQAWAGNGLGAA    105
KacT4     K-KGTKKVAGFYSLATGSVNHTEATGSL-RRNMP-DPIPVIILARLAVDVSLHGKGVGAD    106
                 : .   .   ..      .              . *  *.:      .* .

TacT1     LLHDAVLRCYRVAENIGVRAIMVHALTEEAKNFYIHHGFKSSQTOQRTLFL-----RLPQ    161
TacT2     LVRDAGLRVIQVAETIGIRGMLVHALSDEAREFYQRVGEVPSPMDPMMLMV-----TLGD    158
TacT3     LLLSAGKRCIQAALQVGGVALLIDAKNKOVCDWYKGFGAVPLNDOPLSLLL-----SFKT    164
AtaT      LVAHAMNVVWSASLAVGIHGLFVEALNEKAHTFYKSLGFIPLVGENENALFF----PTKS    166
AtaT2     LLKDAVYRTMSIAQQVGVRALIVHALDDSVRNFYLKYAFVPSPFQSLTLLY-----PITL    160
ItaT      LLCDFFEHVKIIHQALPIKGVYLDAD-PAAINFYARLGFVQLSATPNAFGAVPMFLAIQH    176
KacT      LVAHAMRVVWGASKAVGIYGLFVEALNEKAKAFYLRLGFIQLVDENSNLLFY----PTKS    167
KacT1     LVAHAMRVVWGASKAVGIYGLFVEALNEKAKAFYLRLGFIQLVDENSNLLFY----PTKS    167
KacT2     LVRDAGLRVIQVAETIGIRGMLVHALSDEARDFYLRVGFEPSPMDPMMLMV-----TLRD    157
KacT3     LLKDAIYRTQNIAFQVGVRALAVHALNEEVKCFYTRFGFEPSIVNTLTLLF-----PIKV    160
KacT4     LLHDAVLRCYRVAENIGVRAIMVHALTEEAKGFYAHHGFKASQTHERTLFL-----KLP-    160
          *:             :   .: :.*    .  :*   .

TacT1     -----------    161
TacT2     LVESV------    163
TacT3     LYAALSASGRL    175
AtaT      IELLFTQSD--    175
AtaT2     E----------    161
ItaT      ILAA-------    180
KacT      IEQLFTDDES-    177
KacT1     IEQLFTDDES-    177
KacT2     LVNA-------    161
KacT3     -----------    160
KacT4     -----------    160
```

Figure S3. Multiple sequence alignment of GNAT toxins (n = 11) with their respective amino acid residues that were involved in toxin-antitoxin neutralization. The experimentally-validated amino acid residues that were involved in toxin-antitoxin interaction were marked in green, while the identical active amino acid residues that observed in KacT1-4 were marked in yellow.

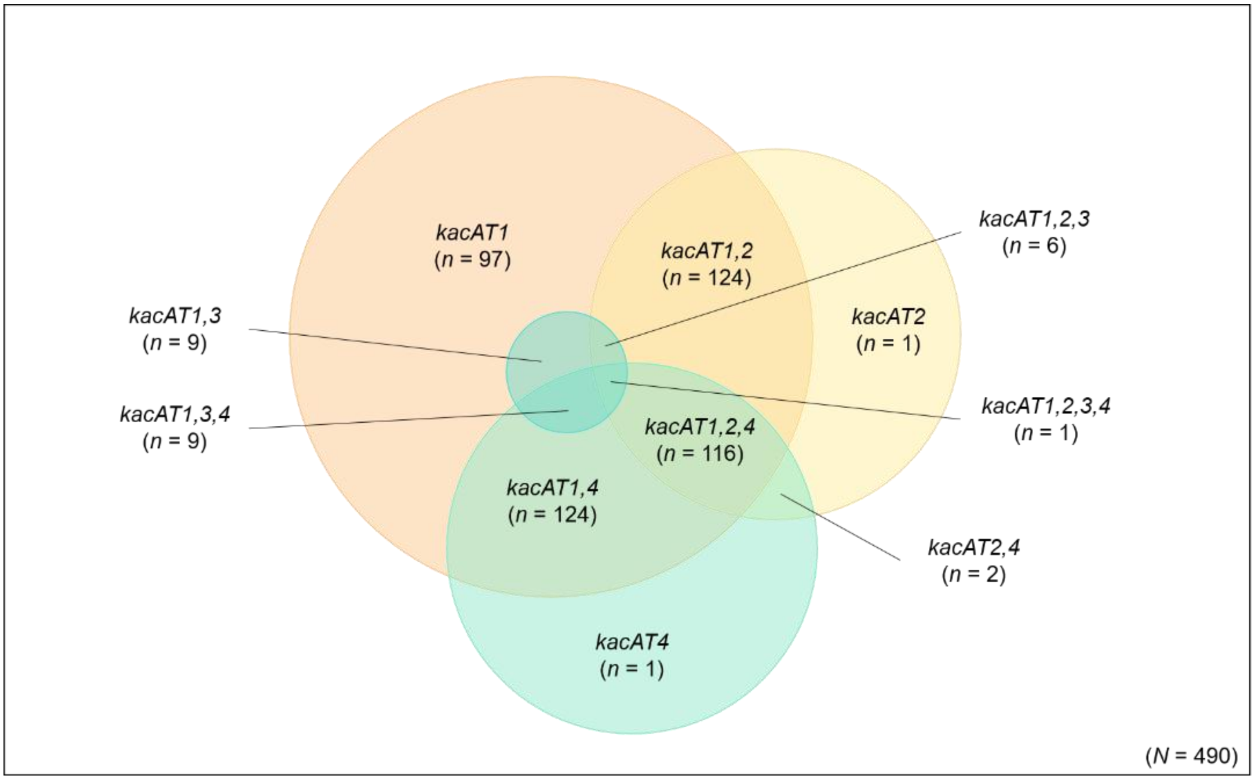

Figure S4. Venn diagram showing the existence of multiple *kacAT* TA loci of different clades in a K. pneumoniae strain. 490 completely sequenced *K. pneumoniae* strains were analyzed. The details for this Venn diagram can be found in **Table S2** and **Table S5**.

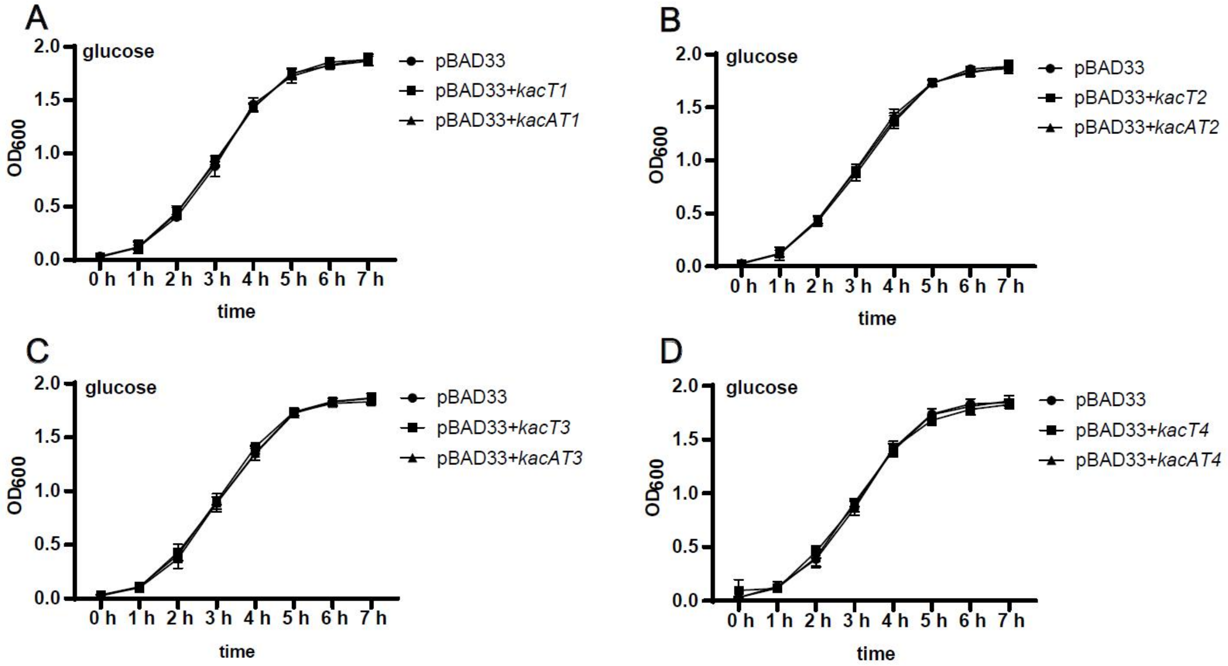

Figure S5. Growth curves of *K. pneumoniae* HS11286-RR2Δ(*kacATkacAT2)* carrying different pBAD33 combinations, induced with glucose.

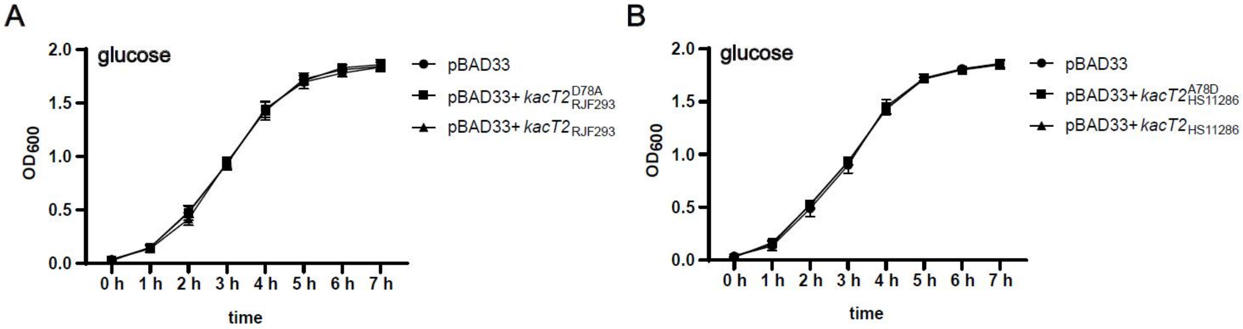

Figure S6. The effect of the amino acid difference between the *K. pneumoniae* GNAT toxins $KacT2_{RJF293}$ and $KacT2_{HS11286}$ on bacterial growth. (**A-B**) Growth curves of *K. pneumoniae* HS11286-RR2Δ*(kacATkacAT2)* with the plasmid containing the wildtype $KacT2_{RJF293}$, the point-mutated $KacT2^{D78A}_{RJF293}$, the wildtype $KacT2_{HS11286}$, or the point-mutated $KacT2^{A78D}_{HS11286}$, respectively.

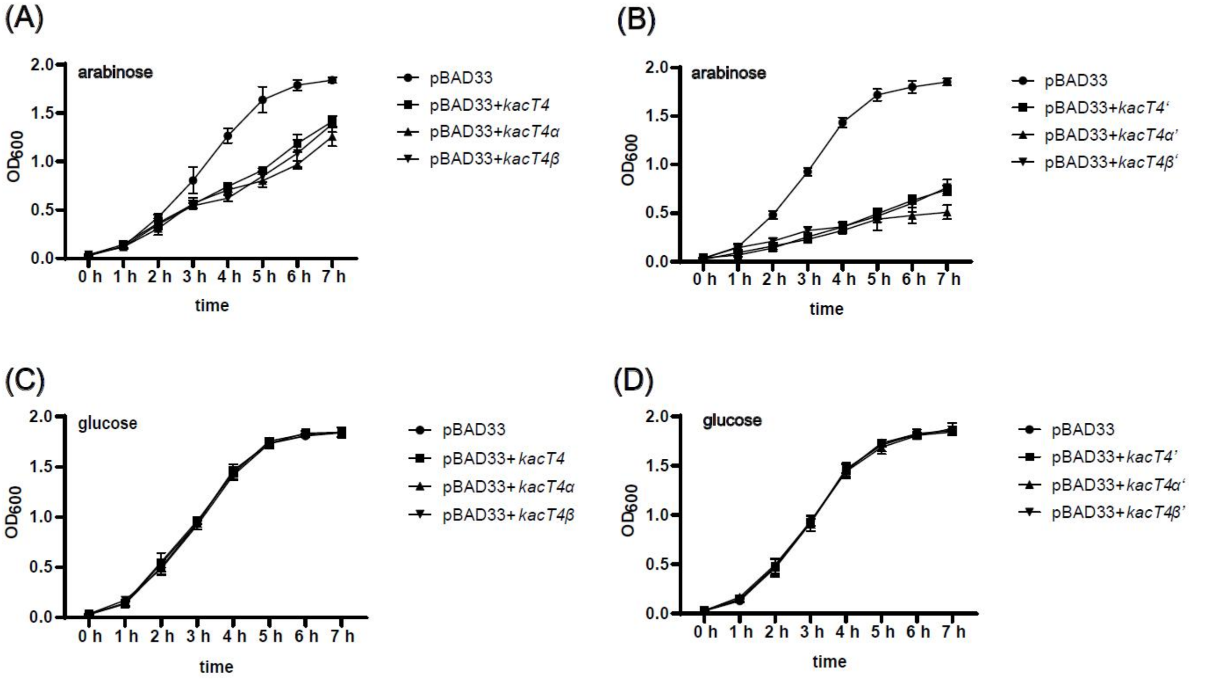

Figure S7. Growth assays to assess the toxicity of the GNAT *protein* KacT4'.**(A-D)** Growth curve of *K. pneumoniae* HS11286-RR2Δ(*kacATkacAT2)* carrying different KacT4s. The strains were incubated in LB broth that contains **(A-B)** arabinose or **(C-D)** glucose at 37°C. The KacT4' represents the optimized KacT4, in which the start codon GUG was replaced by AUG. Optical density at 600 nm was measured at 60-min intervals. Data are presented as the mean ± standard deviation of three independent experiments.

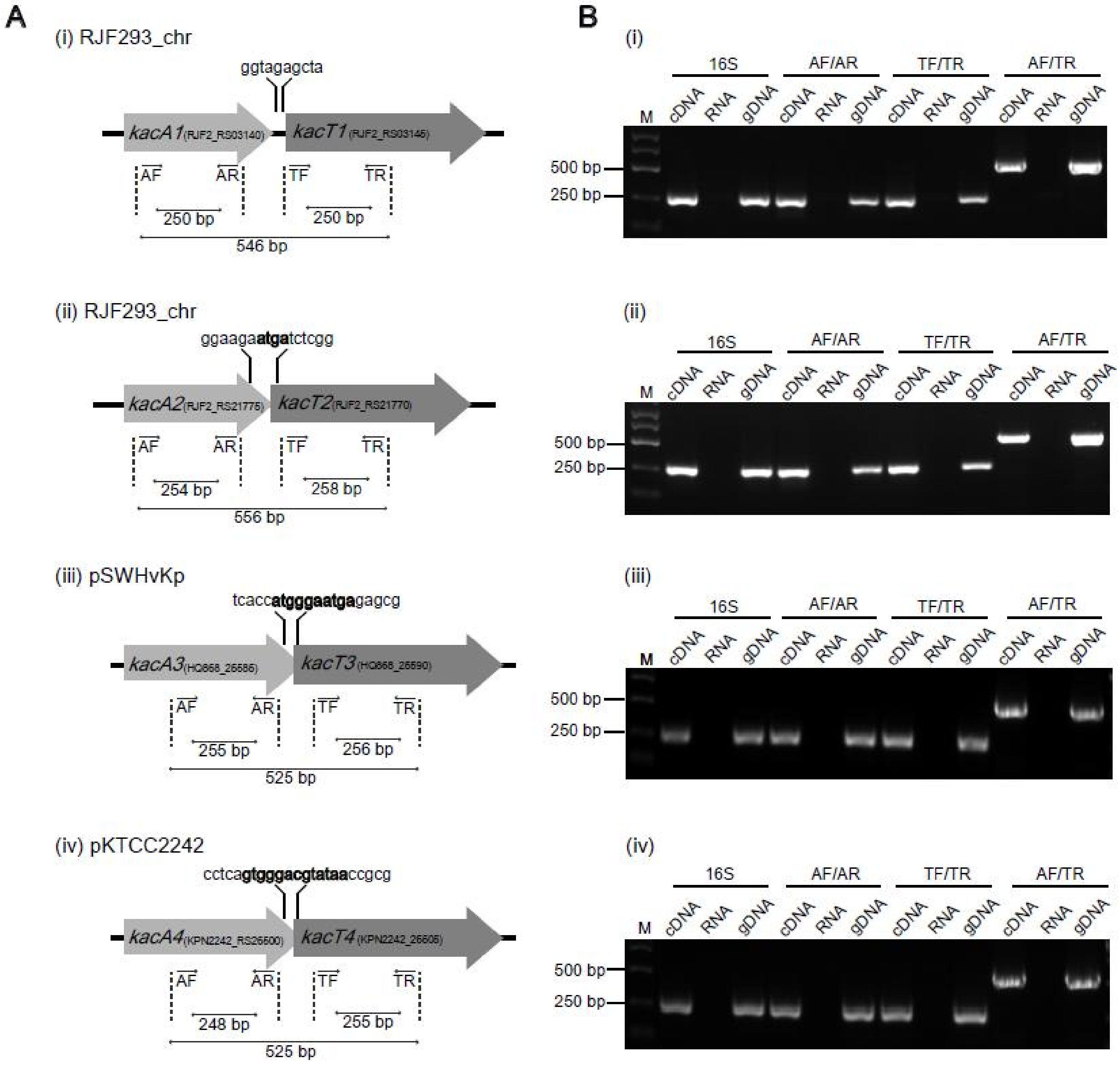

Figure S8. **(A)** Genetic organization and **(B)** co-transcription of GNAT-RHH TA operon. RT-PCR confirmed that the four GNAT-RHH TA loci (*kacAT*1-4) from different phylogenetic clades in *K. pneumoniae* are co-transcripted. The primers used for PCR are listed in **Table S4**.

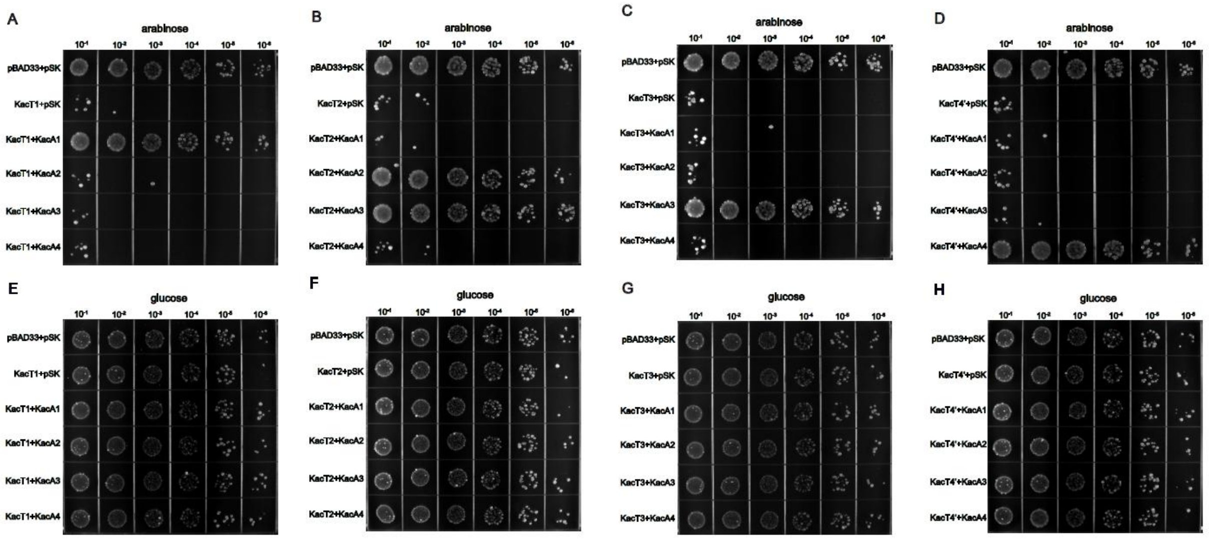

Figure S9. Cross-interaction among different GNAT-RHH TA modules in *K. pneumoniae*. Growth of serially-diluted *K. pneumoniae* HS11286-RR2Δ(*kacATkacAT2)* carrying the different pBAD33-toxin and pSK-antitoxin combinations plated on LB agar supplemented with **(A-D)** arabinose and **(E-H)** glucose. Cross-interaction between the GNAT toxins **(A, E)** KacT1, **(B, F)** KacT2, **(C, G)** KacT3, **(D, H)** and KacT4' with their cognate and non-cognate RHH antitoxins were examined individually. The LB plates were cultured at 37°C overnight.

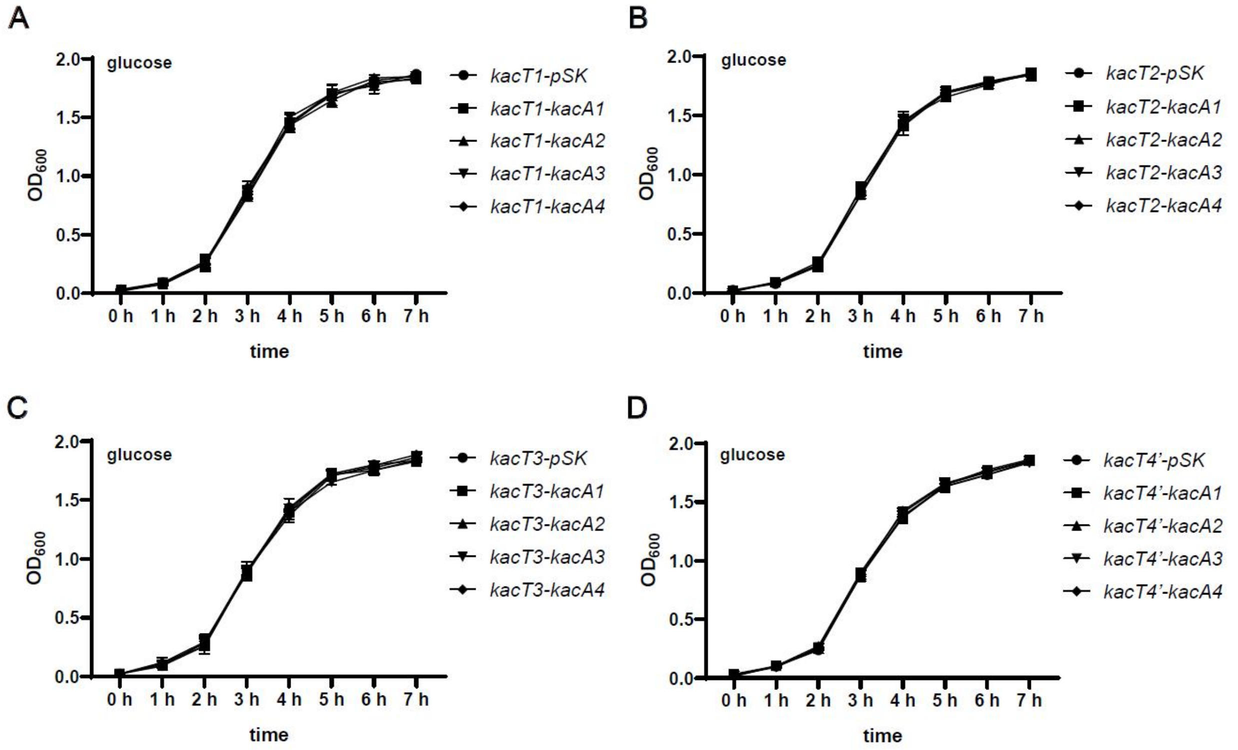

Figure S10. Growth curve of *K. pneumoniae* HS11286-RR2Δ(*kacATkacAT2)* strains containing different pBAD33-toxin and pSK-antitoxin combinations. The strains were incubated in LB broth at 37°C, supplemented with a final concentration of 0.2% glucose. They were the controls for the strains incubated in LB broth supplemented with arabinose (**Fig. 6**). Optical density at 600 nm was measured at 60-min intervals. Data are presented as the mean ± standard deviation of three independent experiments.

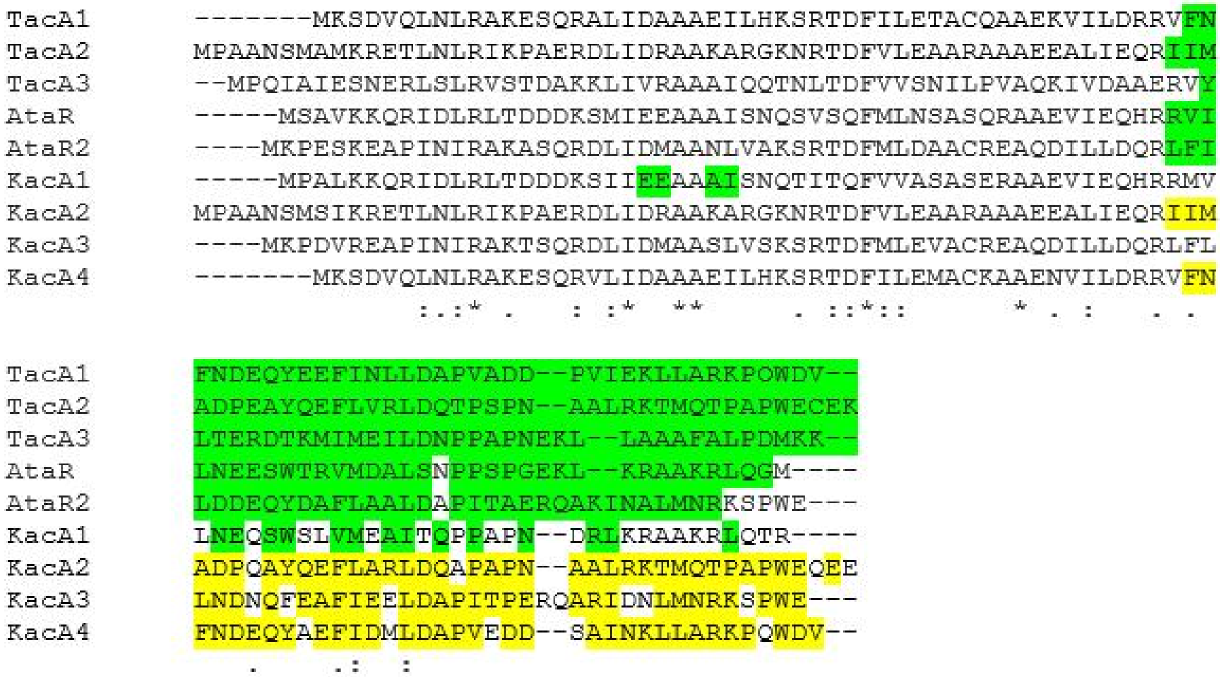

Figure S11. Multiple sequence alignment of amino acid sequence between various RHH antitoxin proteins of *K. pneumoniae*. The experimentally-validated amino acid residues that were involved in toxin-antitoxin interaction were marked in green, while the identical active amino acid residues observed in KacT2-4 and KacA 2-4 were marked in yellow.

REFERENCES

1. Minh BQ, Schmidt HA, Chernomor O, Schrempf D, Woodhams MD, von Haeseler A, Lanfear R. 2020. IQ-TREE 2: New Models and Efficient Methods for Phylogenetic Inference in the Genomic Era. Mol Biol Evol 37:1530–1534.

2. Letunic I, Bork P. 2021. Interactive Tree Of Life (iTOL) v5: an online tool for phylogenetic tree display and annotation. Nucleic Acids Res https://doi.org/10.1093/nar/gkab301.
